# Dietary Change during Pregnancy and Women’s Reasons for Change

**DOI:** 10.3390/nu10081032

**Published:** 2018-08-08

**Authors:** Laura E. Forbes, Jocelyn E. Graham, Casey Berglund, Rhonda C. Bell

**Affiliations:** 1 Department of Family Relations and Applied Nutrition, University of Guelph, 50 Stone Rd., Guelph, ON N1G 2W1, Canada; 2 Department of Agricultural, Food and Nutritional Science, University of Alberta, 116 St. and 85 Ave., Edmonton, AB T6G 2R3, Canada; joc.graham@gmail.com (J.E.G.); caseyburglund@gmail.com (C.B.); Rhonda.Bell@ualberta.ca (R.C.B.)

**Keywords:** pregnancy, nutrition, diet, female, food habits

## Abstract

Women often make dietary changes during pregnancy; however, dietary modifications and reasons for changes are not well studied. We aimed to describe the dietary changes made during pregnancy, describe reasons for dietary changes, and determine what changes aligned with recommendations. Pregnant women (*n* = 379) recruited to the Alberta Pregnancy Outcomes and Nutrition (APrON) study in 2009/2010 completed a questionnaire in which they described dietary changes made during pregnancy and reasons for those changes. Changes and reasons were coded into categories. Women commonly reported increasing their intake of milk products, fruit, and sweet items and commonly decreased or eliminated intake of caffeine, alcohol, and meats. Women frequently reduced intake of foods for the baby’s health and increased foods to satisfy cravings. Changes made commonly aligned with recommendations for caffeine, alcohol intake, food safety, milk and alternatives, and fruit. Changes contrary to recommendations were common for fish and meats. The dietary changes women make during pregnancy appear to reflect women’s efforts to balance physiological changes accompanying pregnancy with the desire for healthy pregnancy outcomes. Understanding the reasons behind dietary change during pregnancy will help researchers and health professionals design effective strategies and public health messages to promote healthier pregnancies.

## 1. Introduction

A healthy, balanced diet during pregnancy is essential to support optimal growth and development of the fetus and the physiological changes that occur in the mother. Fundamental aspects of healthy dietary behaviors during pregnancy include consuming foods that contain optimal amounts of energy as well as macro and micronutrients, achieving appropriate weight gain, adhering to general and pregnancy-specific food safety recommendations, and avoiding ingestion of harmful substances [1,2]. Previous studies have shown that if such behaviors are not adopted, there is an increased risk of adverse pregnancy outcomes including low birth weight [3], preeclampsia [4], pre-term birth [5], and neurodevelopmental problems such as fetal alcohol spectrum disorder [6].

Health Canada and The Public Health Agency of Canada provide several dietary recommendations to help women meet their increased caloric and nutrient needs (Table 1). Additional recommendations include increasing water intake and avoiding foods associated with food-borne illnesses such as undercooked fish and meat, raw eggs, unpasteurized products, and raw sprouts [2,7,8]. Although these guidelines exist to help women select a healthy diet, the extent to which women change their diets to meet pregnancy-related guidelines is unknown.

During pregnancy, motivation for eating a healthy diet may change relative to the non-pregnant state as women prepare for motherhood and consider the impact of their dietary intake on the baby’s health [10]. Personal values and beliefs about nutrition in pregnancy, advice from health professionals, and physical and physiological changes may interact with determinants of eating behaviors present in the non-pregnant state (e.g., personal preferences, time, money) to change diet-related behaviors [11,12,13]. Although most women are aware that healthy eating is important during pregnancy, women may lack knowledge of specific dietary recommendations or may not have the skills required to improve their diet [14]. Healthy eating may also be challenging during pregnancy as women face barriers such as food aversions, cravings, nausea, vomiting, tiredness, constipation, hemorrhoids, and heartburn [15]. Women may receive and follow advice from a variety of sources, including health professionals, peers, and educational resources, which influences their choices during pregnancy [16].

While several international studies have assessed diet before and during pregnancy [17,18,19], these studies have not examined reasons why women may be motivated to make such changes. Understanding factors that motivate or deter pregnant women from making dietary changes is important for devising appropriate means to promote healthy eating behaviors in this population. Therefore, the objectives of this study were to: (1) describe the dietary changes women report making during pregnancy; (2) describe why women made these dietary changes; and (3) determine what changes women make that align with prenatal nutrition recommendations and what motivates them to make these changes.

## 2. Materials and Methods

The present study is a cross-sectional analysis of data collected from the first 400 participants in the Alberta Pregnancy Outcomes and Nutrition (APrON) Study; a prospective cohort study of over 2000 women in Alberta, Canada, with the main aim of examining the link between diet and the mental and physical health of pregnant women and their children [20]. Participants included in this analysis were recruited from medical facilities, local maternity/baby-related businesses, via media campaigns, and the study website. Inclusion criteria were being pregnant at <28 weeks of gestation, over 16 years of age, fluent in English, and residing in the Calgary or Edmonton (Alberta, Canada) areas. Questionnaires used in this study were completed by participants at their first visit with the APrON team, at an average gestational age of 18 weeks gestation (range: 4–34 weeks).

Ethical approval was obtained from the Human Research Ethics Boards at the University of Alberta (project identification code: PRO00002954) and the University of Calgary (project identification code: REB14-1702_REN4). All participants provided written informed consent prior to data collection.

Maternal demographics information was collected by a questionnaire and included women’s current age, pre-pregnancy weight, self-reported gestational age, ethnicity, level of education, marital status, and household income. Height was measured using a digital stadiometer (Charder HM200P Portstad Portable Stadiometer, Charder, Taichung City, Taiwan) and was used with self-reported pre-pregnancy weight to calculate pre-pregnancy body mass index (BMI).

Dietary changes made were assessed using a Dietary Changes Questionnaire designed for the current study (see supplementary materials Table S1 for a copy of the questionnaire). The completed questionnaire was examined for face validity by four prenatal nutrition experts and two researchers with expertise in nutrition survey development. The Dietary Changes Questionnaire was an open-ended survey that asked participants to use their own words to describe the changes they made to their diet since becoming pregnant by listing all foods, beverages, and supplements that they had decreased, eliminated, increased, or added to their diets. For each of the items changed, participants were asked to include the frequency of consumption before and during pregnancy, the normal serving size, and the reason for the change. Sample answers were provided for each question as a guide. Because of the self-reported nature of this questionnaire, the information gathered reflects women’s perceptions of the changes they made and their beliefs about why changes were made.

Answers from completed questionnaires were entered into an Excel spreadsheet (Microsoft Excel, version 12.3.0, 2008, Microsoft, Redmond, WA, USA) and were analyzed using content analysis, a hybrid qualitative and quantitative approach involving examining text for themes or categories, and quantifying the appearance of those categories in the dataset [21]. Individual foods that women reported changing were grouped into categories. Foods were grouped according to the four food groups in the Eating Well with Canada’s Food Guide (EWCFG) [9]. Foods that fit into two or more EWCFG food groups were assigned to a “Mixed Dish” group (e.g., pizza, casseroles). Foods that did not fit with any of the EWCFG food groups were assigned to an “Other Foods” group, which consisted primarily of foods high in salt, sugar, and/or fat and low in nutrients. This Other Foods group was further subdivided into sweet (e.g., candy, chocolate), savory (e.g., pickles, potato chips), spicy (e.g., salsa, hot sauce), and miscellaneous (e.g., gum, mustard) foods. Beverages that were part of a food group according to EWCFG, such as 100% fruit juice and milk, were included in their respective groups. Those not considered part of EWCFG (e.g., water, coffee, tea, alcoholic beverages) were placed into a “Beverages” group.

When analyzing the reasons women gave for making dietary changes in the Dietary Changes Questionnaire, responses were coded into categories, and the number of women reporting responses in each category was counted. Categories and examples of women’s reported reasons included: baby’s health (better for baby, worried about effect on baby); concern (avoiding caffeine, safety risk); aversion (does not taste good, not appealing); nausea (makes me nauseous, nausea); craving (craving sweets, satisfies my craving); nutrient (upping calcium, for omega-3s); to be healthy (good for me, healthier snack); enjoyment (tastes good, enjoy the saltiness); decrease illness (calms my stomach, helps with nausea); other (easy to eat, just because). When more than one reason was listed for a food item, each reason was coded separately. The validity of the analysis was improved by having all responses coded by two of the researchers (J.E.G., L.E.F.) working independently; any disagreements in coding were resolved by consensus.

Maternal age, pre-pregnancy BMI, and weeks of gestation are presented as mean, standard deviation, and range; additional demographic information is presented as the proportion of women reporting a particular answer. Frequencies of food items decreased, eliminated, increased, or added to the diet and the reasons reported for making the change were calculated. Differences in the changes made between women with different demographic characteristics (first vs. second or third trimester, first vs. second+ pregnancy, normal weight vs. overweight or obese and low vs. high income) were examined using *t*-tests to examine differences in the total number of changes and chi-square tests to examine differences in the frequencies of making individual changes. Data analysis was performed using PASW 18.0 (SPSS Inc., 2009, Chicago, IL, USA).

## 3. Results

### 3.1. Demographics

All participants who had completed both the demographics and dietary changes questionnaire were included in this analysis (*n* = 379). The mean age of participants was 31 years (Standard Deviation (SD): 4.1 years) and, on average, they had a healthy pre-pregnancy BMI (Table 2). Most women were Caucasian, had achieved an education level above a high school diploma, and reported an annual household income of greater than CAD70,000.

### 3.2. Dietary Changes

Women reported making an average of six changes to their diets (median: 6, range: 1–19). Women reported increasing or adding foods in the Vegetables and Fruit, Grain Products, Milk and Alternatives, and Other Food groups more frequently than they reported decreasing or eliminating these foods (Table 3). Within the Vegetable and Fruit group, the increase came primarily from increasing fruit intake; 25% reported increasing fruit consumption and only 1% reported decreasing fruit intake, whereas, for vegetables, 13% of women reported increasing consumption and 13% reported decreasing or eliminating a vegetable. Women reported decreasing or eliminating foods from the Meats and Alternatives group more frequently than they reported increasing foods from this group.

In the Other Foods category, participants reported increasing the consumption of sweet foods (16.3%) more frequently than savory (11.3%) or spicy (1.3%) foods. When sweet foods and sweet beverages were combined, 25.1% of participants increased or added sweet items to their diets. More than half of participants reported eliminating a food item classified in the “Miscellaneous” category; these items included chewing gum, artificial sweeteners, garlic, and salad dressing. Beverage intake changed substantially during pregnancy as alcoholic beverages, coffee, and tea were frequently decreased or eliminated from the diet.

Differences in changes made were compared relative to key characteristics of the participants. Women who completed the questionnaire during their first trimester were more likely to report increasing their intake of grain products (29%) compared to those in their second or third trimesters (20%) (*p* = 0.01). Women pregnant with their first child made more changes on average (mean: 6.4 for primiparous vs. 5.5 for multiparous women, *p* < 0.01) and were more likely to report increasing their intake of vegetables and fruit (*p* < 0.001) and decreasing their intake of sweets (*p* = 0.05) compared to women who had other children. Women in the normal pre-pregnancy BMI category were more likely to increase consumption of meats and alternatives compared to those in the overweight or obese category (*p* = 0.03). Women whose household income was less than CAD70,000 per year were more likely to decrease their intakes of grain products compared to women with an income over CAD70,000 (*p* < 0.01).

### 3.3. Reasons for Changing Dietary Intake during Pregnancy

The most common reasons cited for reducing or eliminating specific foods or groups of foods were health of the baby, concern, aversions, and nausea (Table 4). Cravings, nutritional content, health, enjoyment, and to decrease illness were the most frequently reported reasons to increase or add new food items to the diet. Foods consumed for a specific nutrient included nuts and seeds for protein (10.3%), cereal for fiber (9.2%), and fish for omega 3 fatty acids (4.6%). Items such as starches (26.8%), sweet foods (19.6%), and soda (12.5%), were commonly increased or added to the diet to help decrease illnesses including constipation, upset stomach, and heartburn.

### 3.4. Making Changes Aligning with Prenatal Nutrition Recommendations

The most common changes women made that were aligned with dietary recommendations included decreasing caffeine intake (77%), eliminating alcohol intake (53%), following food safety recommendations (i.e., eliminating soft cheese, raw fish or sushi, undercooked meat, unpasteurized milk products, raw or undercooked eggs) (53%), increasing milk and alternative intake (49%), and increasing vegetables and fruit intake (40%). Relatively few women made changes that would bring them in closer alignment with the following recommendations: increasing cooked fish intake (4% increased and 15% decreased intake of cooked fish) and increasing meat and alternative intake (21% increased and 30% decreased intake of meats and alternatives that met food safety recommendations for pregnancy).

The most common reasons women reported for making changes to meet caffeine, alcohol, and food safety recommendations were the baby’s health and concern. The primary reason women increased their intake of milk and alternatives was for the nutrient content, followed by enjoyment and craving. Women’s primary reasons for increasing their vegetable and fruit intake were for cravings, enjoyment, and for a nutrient (most commonly fiber).

Women who increased their intake of meats and alternatives most commonly reported making this change to satisfy a craving or for a particular nutrient (most commonly protein-only three women mentioned iron). Those who decreased their intake of meats and alternatives most commonly reported that the change was due to aversions or nausea. The most common reason women increased their cooked fish intake was the omega-3 fatty acid content. The main reason women reported for decreasing or avoiding cooked fish was to avoid mercury contamination. 

While many women increased intake of folate containing foods during pregnancy (i.e., fruit, grain products made with enriched flour), the most common reasons for increasing these foods were cravings, enjoyment and, for grain products, to decrease illness. No women indicated that they increased their intake of grains or vegetables and fruit in their diet to improve their intake of folate or folic acid.

## 4. Discussion

The patterns of dietary change during pregnancy reported in this study indicate that women understand and report reducing intake of foods that could harm their pregnancy, but do not increase their intake of foods that provide important nutrients required for pregnancy. This suggests that women do not prioritize having a nutrient-dense diet when making dietary changes, which may result in suboptimal intakes of nutrients that are key for prenatal development.

### 4.1. Dietary Changes

Earlier studies conducted in the United States of America (USA) [22] and the United Kingdom (UK) [17] documented a number of similar dietary changes as the current study. For example, Hook [22] noted that 50% of women reported increasing their milk consumption while pregnant. Aversion to meats was reported by 26% of participants and 36% reported craving sweet foods. Crozier and colleagues [17] administered food frequency questionnaires prior to and during pregnancy and reported that women increased their consumption of dairy products and sweet foods and beverages. Regular intake of foods and beverages high in sugar by non-pregnant individuals has been associated with poor diet quality [23] and, during pregnancy, high sugar foods may promote excess calorie intake and excess gestational weight gain [24]. While there are no specific recommendations for sugar consumption during pregnancy, the World Health Organization (WHO) recommends that adults consume no more than 10% of their energy from free sugars [25]. Women may benefit from guidance on building strategies to limit intake of high sugar foods and beverages by managing cravings and regularly making healthier substitutions.

### 4.2. Reasons for Changing Dietary Intake During Pregnancy

This study extends our knowledge of dietary changes during pregnancy by documenting women’s reasons for undertaking specific changes. Overall, women who reported increasing their intake of food items did so because of a craving, while they decreased their intake of foods to promote the health of the baby. Previous studies have shown that food cravings are a strong motivator for increasing food intake among pregnant women [26] and that pregnant women consume fewer foods that could pose a safety risk than non-pregnant women [27]. Together, these results suggest that there are a number of factors that affect intake among pregnant women and that women experience multiple, and sometimes contradictory, influences that may affect food intake during pregnancy. Health care providers should help women explore these multiple factors when working with clients to identify feasible and practical strategies to optimize food intake for pregnancy. The present study’s simultaneous examination of women’s behaviors and motivations is unique and provides vital information that may help to establish target areas and strategies for intervention in the future.

### 4.3. Making Changes Aligning with Prenatal Nutrition Recommendations

Our analysis of the changes women made that align with nutrition recommendations suggests which recommendations are known and salient to women and what factors motivate women to make these changes. The findings for caffeine, alcohol, and foods with safety concerns indicate that women are aware of recommendations to reduce their intake and that fear of harming the baby is an important factor that women consider when making these changes. Women’s reported reasons for increasing intake of milk and alternatives and fruit suggest that women are likely to add healthy foods to their diets if the foods are also craved or enjoyable to consume.

Many women reported decreasing intake of both cooked fish and meats and alternatives; these changes are contrary to recommendations. These findings indicate that positive dietary changes may be difficult to make if recommendations are complicated as with fish (women are recommended to eat fish but to avoid mercury-containing fish) or if physiological symptoms of pregnancy such as nausea and aversions reduce the intake of recommended foods. Previous studies have shown that women struggle to understand recommendations for fish intake during pregnancy [28] and that aversions to meats are common during this time [15]. Women may also rely on supplemental sources of the key nutrients in fish and meat to meet recommendations. A previous analysis of data from the APrON study showed that in the first trimester, 11% of women reported taking essential fatty acid supplements (primarily omega 3 or fish oil supplements) and 91% of participants used iron-containing supplements [29]. Thus, relying on supplemental sources of these important nutrients may be more achievable when making dietary changes is challenging.

### 4.4. Strengths and Limitations

This study used an open-ended questionnaire, which can be considered a strength as it allowed participants to include any type of food or beverage that was altered in the diet and reason(s) for these changes. This questionnaire format allowed women to use their own words in describing their experience but could be prone to limitations associated with social acceptability, willingness to provide information, and memory. One possible limitation of the open-ended questionnaire was that examples were provided as a guide and this may have contributed to recall bias. In particular, decreasing coffee intake was an example provided in the questionnaire and the high number of women reporting decreasing or eliminating coffee in this study may be partially reflective of recall bias. Women completed the dietary changes questionnaire between 4 and 34 weeks of pregnancy. This could be a seen as a limitation due to inconsistent timing of the questionnaire relative to gestational age among participants. However, our analysis showed few differences in responses between women who completed the questionnaire in their first vs. second or third trimester. A strength of this study is the robust sample size which resulted in data saturation for the descriptions of changes women made and the reasons for these changes. The high socioeconomic status of our participants is a limitation indicative of volunteer bias and may provide a “best case” scenario for healthy eating behaviors in pregnancy as the women may have the educational, financial and social resources necessary to support a healthy diet [30]. Future studies should target women across a broader range of socioeconomic status to provide a more comprehensive view of dietary changes made during pregnancy and the reasons for those changes.

## 5. Conclusions

This study has described what changes women make to their diets during pregnancy and their reasons for making these changes. In addition, it has identified which changes women make that align with dietary recommendations and which dietary recommendations may be more challenging for women to achieve. Examination of the women’s reported reasons behind their dietary changes suggest that the health of the baby is often cited and that women balance a range of other factors such as cravings and nausea when deciding what changes to make. This study also shows that women frequently make changes to reduce intake of compounds that could harm their pregnancy, but are less likely to increase their intake of foods that provide key nutrients. These findings give insight into how and why women make changes to their diets during pregnancy and researchers and practitioners can use this information to inform the development of practical, feasible interventions to help women optimize their dietary intake during pregnancy.

## Figures and Tables

**Table 1:** Dietary recommendations for pregnancy.

Dietary Component	Recommendation
EWCFG ^1^	Add 2–3 servings from any food group during the second and third trimester in addition to the daily recommendation ^1,2,3^
Vegetables and Fruit	7–8 servings
Grain Products	6–7 servings
Milk and Alternatives	2 servings
Meat and Alternatives	2 servings
Cooked fish	150 g/week ^1^
Calcium	1000 mg before and during pregnancy ^3^
Iron	18 mg before pregnancy; 27 mg during pregnancy ^3^
Folic acid	400 μg before pregnancy; 600 μg during pregnancy ^3^
Caffeine	Maximum 300 mg/day ^3^
Alcohol	No alcohol ^3^

^1^ Recommendations from Health Canada [8]; ^2^ Eating Well with Canada’s Food Guide [9]; ^3^ Recommendations from Public Health Agency of Canada [2,7]. EWCFG: Eating Well with Canada’s Food Guide.

**Table 2:** Characteristics of pregnant women in the Alberta Pregnancy Outcomes and Nutrition (APrON) Study included in this analysis. BMI: body mass index.

Characteristic	Mean ± SD (Range) or *n* (%) *n* = 379
Maternal age	31.28 ± 4.12 (18–43)
Pre-pregnancy BMI (kg/m^2^)	24.21 ± 4.57 (16–43)
Weeks of gestation at time of study	18.02 ± 5.58 (4–34)
Trimester at time of study	
First trimester	83 (21.9)
Second trimester	284 (74.9)
Third trimester	4 (1.1)
Race/ethnicity	
Caucasian	335 (88.4)
Other	37 (9.8)
Education	
High school or less	32 (8.4)
Trade or post-secondary	341 (90.0)
Marital status	
Married or common law	359 (94.7)
Divorced or single	13 (3.4)
Household income ($)	
>70,000	305 (80.5)
<69,000	60 (15.8)
Smoking in household	
No one smokes	347 (91.6)
One person or more smokes	7 (1.8)

**Table 3:** Proportion of women who reported dietary changes during their pregnancy.

	Dietary Change *n* (%)			
Item Category	Decreased	Eliminated	Increased	Added
Vegetables and Fruit	31 (8.2)	31 (8.2)	127 (33.5)	23 (6.1)
Grain Products	10 (2.6)	14 (3.7)	75 (19.8)	31 (8.2)
Milk and Alternatives	22 (5.8)	70 (18.5)	154 (40.6)	31 (8.2)
Meat and Alternatives	99 (26.1)	208 (54.9)	67 (17.7)	12 (3.2)
Other Foods ^1^	52 (13.7)	44 (11.6)	95 (25.1)	37 (9.8)
Beverages ^2^	208 (54.9)	304 (80.2)	48 (12.7)	30 (7.9)
Mixed Dishes ^3^	11 (2.9)	14 (3.7)	12 (3.2)	6 (1.6)

^1^ Other Food items are those that do not fit in EWCFG groups (e.g., candy, chips/fries, chocolate, gum); ^2^ Beverages includes items that are not included in EWCFG (e.g., alcohol, coffee, tea, sweet drinks, soda); ^3^ Mixed Dishes were defined as foods that are a mixture of food items defined by EWCFG (e.g., pizza, lasagna).

**Table 4:** Proportion of women who reported a specific reason for changing their dietary intake and the three most commonly cited foods associated with that reason.

Reason Foods Commonly Changed	*n* (%) ^1,2^
**Top reasons for decreasing or eliminating intake of foods**	
Baby’s Health	246 (64.9)
Alcohol	163 (66.3)
Coffee	81 (33.0)
Sushi/Raw Fish	62 (25.2)
Concern	181 (47.8)
Coffee	93 (51.4)
Undercooked Meat	48 (26.5)
Tea	37 (20.4)
Aversion	100 (26.4)
Coffee	26 (26.0)
Red Meat	15 (15.0)
Vegetable	14 (14.0)
Nausea	86 (22.7)
Coffee	20 (23.3)
Vegetables	19 (22.1)
Red Meat	13 (15.1)
**Top reasons for adding or increasing intake of foods**	
Craving	197 (52.0)
Sweet Foods ^3^	64 (32.5)
Fruit	55 (27.9)
Milk	32 (16.2)
Nutrient	87 (23.0)
Milk	42 (48.3)
Cheese	13 (14.9)
Yogurt	11 (12.6)
To be Healthy	67 (17.7)
Fruit	11 (16.4)
Milk	10 (14.9)
Vegetables	9 (13.4)
Enjoyment	66 (17.5)
Fruit	22 (33.3)
Sweet Foods	17 (25.8)
Milk	11 (6.7)
Decrease Illness	56 (14.8)
Starches	15 (26.8)
Sweet Foods	11 (19.6)
Soda	7 (12.5)

^1^ Some participants reported more than one reason for changing a food item. The total number of participants (379) was used as the denominator to calculate the % of women reporting a reason; ^2^ Participants could change multiple food items for the same reason. Values refer to the % of women reporting foods changed with the total number of women who reported that specific reason as the denominator; ^3^ Sweet Foods includes desserts, baking, candy, chocolate, ice cream, etc.

## Supplementary Materials

The following is available online at http://www.mdpi.com/2072-6643/10/8/1032/s1, Table S1: Dietary changes made since becoming pregnant.
