# Supplementary material for: Dietary Change during Pregnancy and Women’s Reasons for Change
Source: Nutrients. 2018 Aug 8;10(8):1032. doi: 10.3390/nu10081032 (PMC6115730; doi:10.3390/nu10081032)
Supplement: Supplementary file 1 [file nutrients-10-01032-s001.pdf]

Study ID: __________________

DIETARY CHANGES MADE SINCE BECOMING PREGNANT

The following questions will ask about any **dietary changes** that you have made **since you have become pregnant**. Please include the **number of times** you consumed the food or drink per week or per day, the **amount** of the item you consumed each time, and a reason or reasons to explain **why** the change was made.

1. **Since you have become pregnant**, have you completely **eliminated** any foods or beverages from your diet that you consumed prior to becoming pregnant?

☐ NO (GO TO QUESTION 2)
☐ YES (CONTINUE BELOW WITH 1a)

1a. Please list the food(s) and beverage(s) you completely **eliminated** from your diet, a reason explaining why the change was made, and your consumption of the item(s) **prior** to pregnancy.

FOOD ITEM	REASON FOR ELIMINATION OF FOOD (i.e. for the health of my baby, had no appetite for the food, etc.)	CONSUMPTION OF ITEM **PRIOR** TO PREGNANCY (portion; number of times per week or per day (please specify))	
		**Amount**	**Frequency**
**i.e.** Coffee	Smell made me nauseous	1 cup	2 times /day

2. **Since you have become pregnant**, have you **decreased** your consumption of **any** specific foods or beverages?

☐ NO (GO TO QUESTION 3)
☐ YES (CONTINUE BELOW WITH 2a)

2a. Please list which food(s) and beverage(s) you **decreased** your consumption of. Note the amount and frequency of the food consumed prior to pregnancy and since learning that you were pregnant. Also, provide a brief explanation why the change was made.

FOOD ITEM	CONSUMPTION **PRIOR** TO PREGNANCY (Amount and Frequency per week or per day)		CONSUMPTION **SINCE LEARNING** OF PREGNANCY		REASON FOR DECREASE IN CONSUMPTION
	**Amount**	**Frequency**	**Amount**	**Frequency**	
**i.e.** Tea	1 cup	5 times/week	1 cup	2 times/week	Drank more milk instead of tea

3. **Since you have become pregnant**, have you **introduced** any **brand new** foods or beverages in your diet that you never consumed prior to becoming pregnant?

☐ NO (GO TO QUESTION 4)
☐ YES (CONTINUE BELOW WITH 3a)

3a. Please list the **brand new** food(s) and beverage(s) you **introduced** into your diet, a reason explaining why the change was made, and your consumption of the item(s) since becoming pregnant.

FOOD ITEM	REASON FOR INTRODUCTION OF FOOD	CONSUMPTION OF ITEM SINCE PREGNANCY	
		**Amount**	**Frequency**
**i.e.** Ice cream	Continuous craving for ice cream	1 cup; 5 times/week	

4. **Since you have become pregnant**, have you **increased** your consumption of **any** specific foods or beverages?

□ NO (GO TO QUESTION 5)
□ YES (CONTINUE BELOW WITH 4a)

4a. Please list which food(s) and beverage(s) you **increased** your consumption of. Note the amount and frequency of the food consumed prior to pregnancy and since learning that you were pregnant. Also, provide a brief explanation why the change was made.

FOOD ITEM	CONSUMPTION **BEFORE** PREGNANCY (Amount and Frequency per week or per day)		CONSUMPTION **SINCE LEARNING** OF PREGNANCY		REASON FOR INCREASE IN CONSUMPTION
	**Amount**	**Frequency**	**Amount**	**Frequency**	
**i.e.** Pickles	1 pickle	3 times/week	3 pickles	6 times/week	Craved salt and enjoyed the crunch

5. In the space below, please make any other comments regarding **changes in dietary intake since becoming pregnant** (i.e. Did you combine certain foods that you would not have normally combined? Where there specific times throughout the day when you craved certain foods? Etc.)

6. **Since becoming pregnant (or finding out that you are pregnant)**, did you change your use of supplements?

□ NO (GO TO QUESTION 7)
□ YES (CONTINUE BELOW WITH 6a)

6a. Please list any **prenatal supplements, multivitamins, or single supplements** that you began to take since finding out that you are pregnant (i.e. Centrum Materna, Jamieson Prenatal Natural Source Multivitamin, iron, folate, calcium, vitamin D, Fish Oil (omega 3 fatty acids), etc.)? *Please list the frequency of supplementation and amount of nutrient in each dose for both pre-pregnancy intake and current pregnancy intake.*

SUPPLEMENT	SUPPLEMENTATION **BEFORE** TO PREGNANCY (Amount and Frequency per week or per day)		SUPPLEMENTATION **SINCE LEARNING** OF PREGNANCY		REASON FOR CHANGE
	**Amount**	**Frequency**	**Amount**	**Frequency**	
**i.e.** Iron	None	None	27mg	1 time/day	Iron supplementation was recommended by General Practitioner

7. Please tell us the date that you completed this questionnaire: _____day_____month____year
